# Molecular Docking and In Vitro Evaluation of Violacein-Alginate Beads for Targeted Inhibition of *Staphylococcus aureus* Biofilm Formation

**DOI:** 10.1021/acsomega.5c03106

**Published:** 2025-07-16

**Authors:** Çağdaş Deniz Peri̇z, Seyhan Ulusoy, Neslihan Kaya Kinaytürk

**Affiliations:** 1 Faculty of Engineering and Natural Sciences, Biology Department, Süleyman Demirel University, Isparta 32260, Türkiye; 2 Faculty of Arts and Science, Nanoscience and Nanotechnology Department, Mehmet Akif Ersoy University, Burdur 15100, Türkiye

## Abstract

Violacein, produced by *Chromobacterium violaceum*, exhibits significant antibacterial, antiviral, antifungal, and antioxidant properties. However, its poor aqueous solubility substantially limits its bioavailability. To overcome this constraint, violacein was encapsulated in sodium alginate beads, and its antibiofilm efficacy against *Staphylococcus aureus* was evaluated. Violacein-loaded alginate beads (VIABs) were synthesized and characterized using Fourier-transform infrared spectroscopy, scanning electron microscopy, and ultraviolet–visible spectrophotometry. Molecular docking analysis was further conducted to examine the interactions between violacein and the *icaADBC* proteins (UniProt accession: Q9RQP9, Q9RQP8, Q9RQP7, andQ9RQP6 ), which play a critical role in polysaccharide intercellular adhesin production during biofilm formation. The crude violacein extract displayed antibacterial activity, generating an inhibition zone of 12.3 ± 0.5 mm against *S. aureus*. The average particle sizes of dry alginate beads and violacein-loaded alginate beads were 0.97 ± 0.16 and 0.66 ± 0.11 mm, respectively. VIABs (20–5 mg/mL) significantly suppressed *S. aureus* biofilm formation by 77.4, 67.4, and 46.8%. Molecular docking analysis demonstrated strong binding affinities between violacein and the target proteins. Furthermore, violacein adhered to Lipinski’s Rule of Five, suggesting favorable pharmacokinetic properties. These findings highlight the potential of VIABs as a promising therapeutic and food preservative agent due to their potent antibiofilm activity.

## Graphical abstract

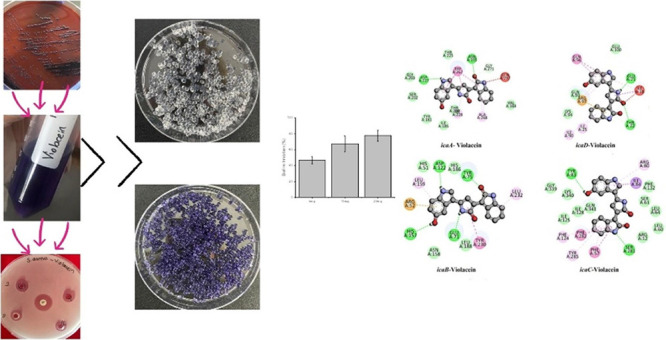

## Introduction

Nature offers a vast array of secondary metabolites with diverse molecular structures that have been utilized in numerous applications across multiple industries, including cosmetics, textiles, food, and healthcare. Microbial pigments, in particular, function as defense mechanisms against diverse environmental stressors, including predation and microbial competition.^1–3^

Among these pigments, violacein—a compound extracted from *Chromobacterium violaceum*—has garnered increasing interest among researchers due to its diverse range of biological activities. Over the past two decades, numerous studies have highlighted the diverse biological activities of this pigment, including antimicrobial, antifungal, anticancer, and antiviral effects.^4,5^ Furthermore, violacein has been shown to exhibit reduced toxicity and enhanced biocompatibility compared to synthetic antibiotic agents.^6–10^

*Staphylococcus aureus* is a prominent opportunistic human pathogen associated with high morbidity and mortality rates.^11^ *S. aureus* poses a significant clinical concern due to its propensity for causing a wide range of infections, largely attributed to its ability to form biofilms and produce toxins.^11–15^ Biofilms are implicated in over 80% of microbial infections and are notoriously resistant to antibiotic treatment.^16–19^ The biofilm matrix acts as a physical barrier, impeding the penetration of drugs into the bacterial community, thus enabling the microbes to resist and diminish the efficacy of conventional antibiotics.^20^ The formation of *S. aureus* biofilms is primarily linked to the biosynthesis of polysaccharide intercellular adhesins (PIA/IcaADBC), which encode three membrane-associated proteins (IcaA, IcaD, and IcaC) and one extracellular protein (IcaB).^21^ PIA serves as the key biomolecule mediating cell aggregation and biofilm maturation. Its production is facilitated by enzymes encoded within the ica operon (icaADBC).^22^

These challenges have sparked considerable interest within the scientific community in developing natural-based therapeutics as safer and more environmentally friendly alternatives to antibiotics. However, microbial and natural pigments are highly susceptible to degradation under physical and chemical stress, resulting in diminished bioactivity and reduced shelf life. Alginate-based delivery systems offer several significant advantages including improved physical and mechanical properties (e.g., swelling, particle size, viscosity).^23^ Additionally, these systems can protect sensitive bioactive components from harsh environmental conditions (such as strong acidity, oxygen, bacteria, UV light).^24^ Sodium alginate (SA), a natural, biodegradable, and biocompatible polysaccharide that is FDA-approved, contributes to improved encapsulation stability through its carboxyl and hydroxyl groups.^25–28^ Moreover, SA readily cross-links with Ca^2+^ ions, forming egg-box structures that enhance water permeability, adsorption capacity, and material stability.^29^

This study aimed to encapsulate violacein within Ca-alginate beads via the ionic gelation technique and to systematically characterize the fabricated beads using FTIR, swelling ratio, and SEM analyses to assess their physicochemical attributes. The antibacterial and antibiofilm properties of violacein-loaded alginate beads were investigated against *S. aureus*. Moreover, molecular docking and ADMET analyses were conducted to unveil the potential of violacein-loaded alginate beads as innovative natural additives in diverse food, pharmaceutical, and cosmeceutical applications.

## Results and Discussion

Violacein extracted from *C. violaceum* (Figure S1) and exhibited antibacterial activity against Gram-positive *S. aureus* at concentrations ranging from 25 to 200 μg/mL, with inhibition zone diameters of 12.3 ± 0.5, 11.0 ± 0.0, 9.3 ± 0.5, and 8.3 ± 0.5 mm, respectively (Table 1, Figure 1a).

**Table 1:** Inhibition Zone Diameters of Violacein (200–25 μg/mL) Extracted from *C. violaceum* ATCC 12472 against *S. aureus*

	inhibition zone diameters (mm)				kanamycin
violacein (μg/mL)	200	100	50	25	30 μg
S. aureus	12.3 ± 0.5	11.0 ± 0	9.3 ± 0.5	8.3 ± 0.5	16.0 ± 0

**Figure 1:**
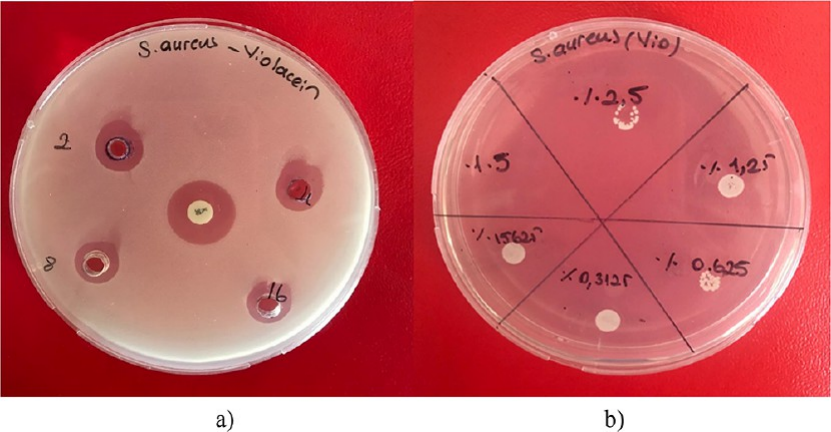
(a) Inhibition zones of violacein at 200 (2), 100 (4), 50 (8), and 25 μg/mL (16) against *S. aureus*. (b) Minimum bactericidal concentration (MBC: 200 μg/mL, 5%) and minimum inhibitory concentration (MIC: 100 μg/mL, 2.5%) of violacein. K: Positive control (kanamycin, 30 μg/mL).

The minimum inhibitory concentration (MIC) for violacein against *S. aureus* was determined to be 100 μg/mL, while the minimum bactericidal concentration (MBC) was 200 μg/mL Figure 1 **b.**

The antibacterial and biofilm-inhibitory properties of violacein against *Staphylococcus aureus* have been reported in prior studies.^8,30–33^ In this study, the minimum inhibitory concentration (MIC) for *S. aureus* was found to be lower than the MIC values reported by Nakamura et al. (2003)^32^ and Nakazato et al. (2019)^33^ for crude violet pigment, which were 15 mg/L and 200 μg/mL, respectively.

The effects of violacein on *S. aureus* biofilm formation were assessed using the crystal violet assay, with spectrophotometric measurements at OD570 nm. The biofilm inhibition rates increased proportionally with rising violacein concentrations (12.5, 25, and 50 μg/mL), yielding inhibition rates of 28.1%, 31.5%, and 50%, respectively.

### Preparation of Alginate and Violacein-Alginate Beads

Alginate and violacein-alginate beads were prepared using the ionic gelation method.^34^ Bead size in both wet and dry states was analyzed using ImageJ software (Figure 2 **a-d**). The average particle sizes of wet alginate and violacein-alginate beads were 2.33 ± 0.27 mm and 1.86 ± 0.21 mm, while those of dry alginate and violacein-alginate beads were 0.97 ± 0.16 mm and 0.66 ± 0.11 mm, respectively. The prepared beads exhibited a uniform, spherical morphology. Violacein-alginate beads displayed a dark violet color similar to that of the violacein pigment. Histogram graphs illustrating the visual appearance and size distribution of the beads in both wet and dry states are presented in Figure 2 **a-d.**

**Figure 2:**
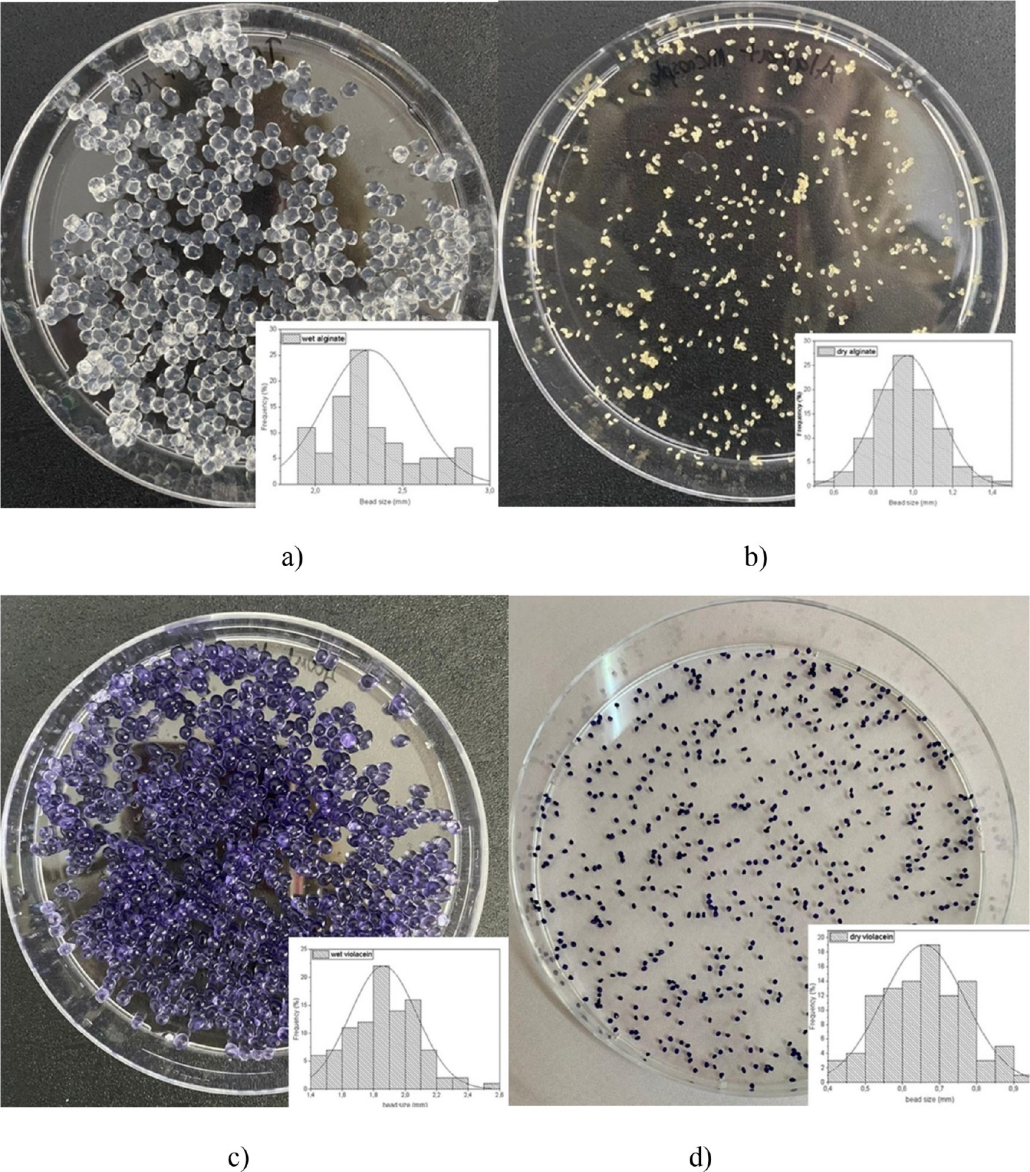
Histogram and digital photographs of alginate (a, b) and violacein-alginate (c, d) beads in wet (a, c) and dry (b, d) states.

Bead size changed significantly after drying, with a mean diameter reduction from 2.33 mm to 0.97 mm for alginate beads (Figure 2 **a-b**) and from 1.86 mm to 0.66 mm for violacein-alginate beads (Figure 2 **c-d**), corresponding to a 2.4–2.8-fold decrease.

SEM images of dried alginate and violacein-alginate beads revealed that alginate beads had a smoother and more spherical surface morphology, whereas violacein incorporation resulted in rougher, more wrinkled surfaces (Figure 3 **a-b**). These variations are attributed to factors such as drying rate, formulation composition, solvent properties, and the nature of the loaded active substance.^35^

**Figure 3:**
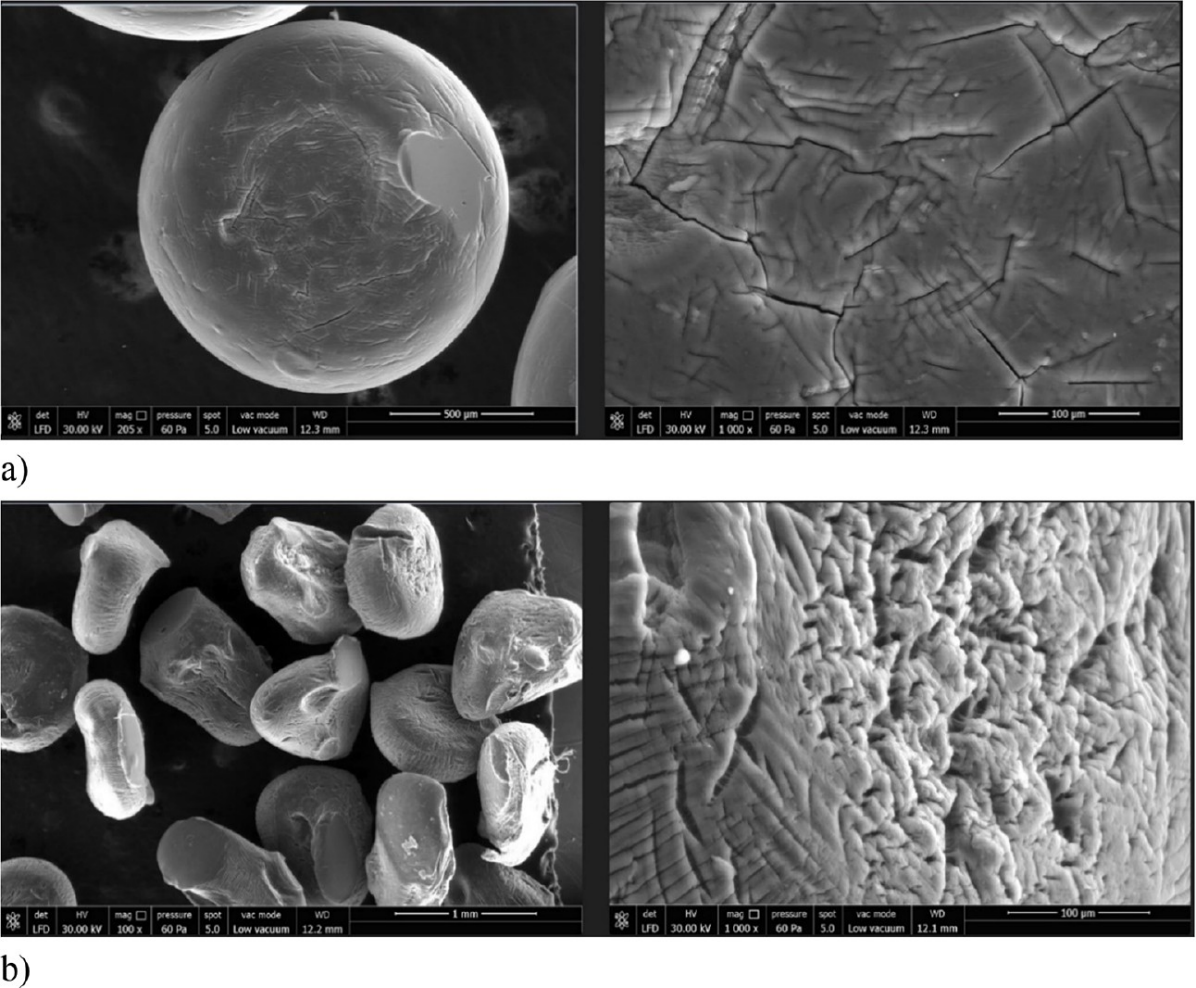
Scanning electron micrographs (SEM) of (a) dry alginate beads and (b) dry violacein-alginate beads at ×1000 magnification.

### Fourier-Transform Infrared Spectroscopy (FTIR) Analysis of Alginate and Violacein-Alginate Bead

To investigate potential interactions between violacein and alginate responsible for observed structural differences, FTIR analysis was conducted.

The FTIR spectra of sodium alginate and alginate beads are presented in Figure 4 **a-b**. The O–H stretching vibrations of sodium alginate were observed between 3000 and 3600 cm^–1^, with a peak at 3291 cm^–1^. The C–H stretching vibrations appeared between 2920 and 2850 cm^–1^, with a peak at 2921 cm^–1^. Characteristic C = O bonds, typical of alginates, were detected between 1600 and 1700 cm^–1^, with a peak at 1594 cm^–1^.^36^ The FTIR spectrum of sodium alginate beads indicated the influence of calcium ions without cross-linking. The vibration at 1586 cm^–1^, attributed to Ca^2+^ cross-linking, and a reduction in peak intensities at 1313 cm^–1^ and 1015 cm^–1^ suggest decreased cross-linking in violacein-alginate beads. The reduction in peak depth suggests weak bonding or masking by dominant bands, indicating interactions between alginate, violacein, and Ca^2+^ ions. This suggests that the hydroxyl (−OH) groups in violacein may form intermolecular hydrogen bonds with alginate molecules and share coordination regions with Ca^2+^.^37^

**Figure 4:**
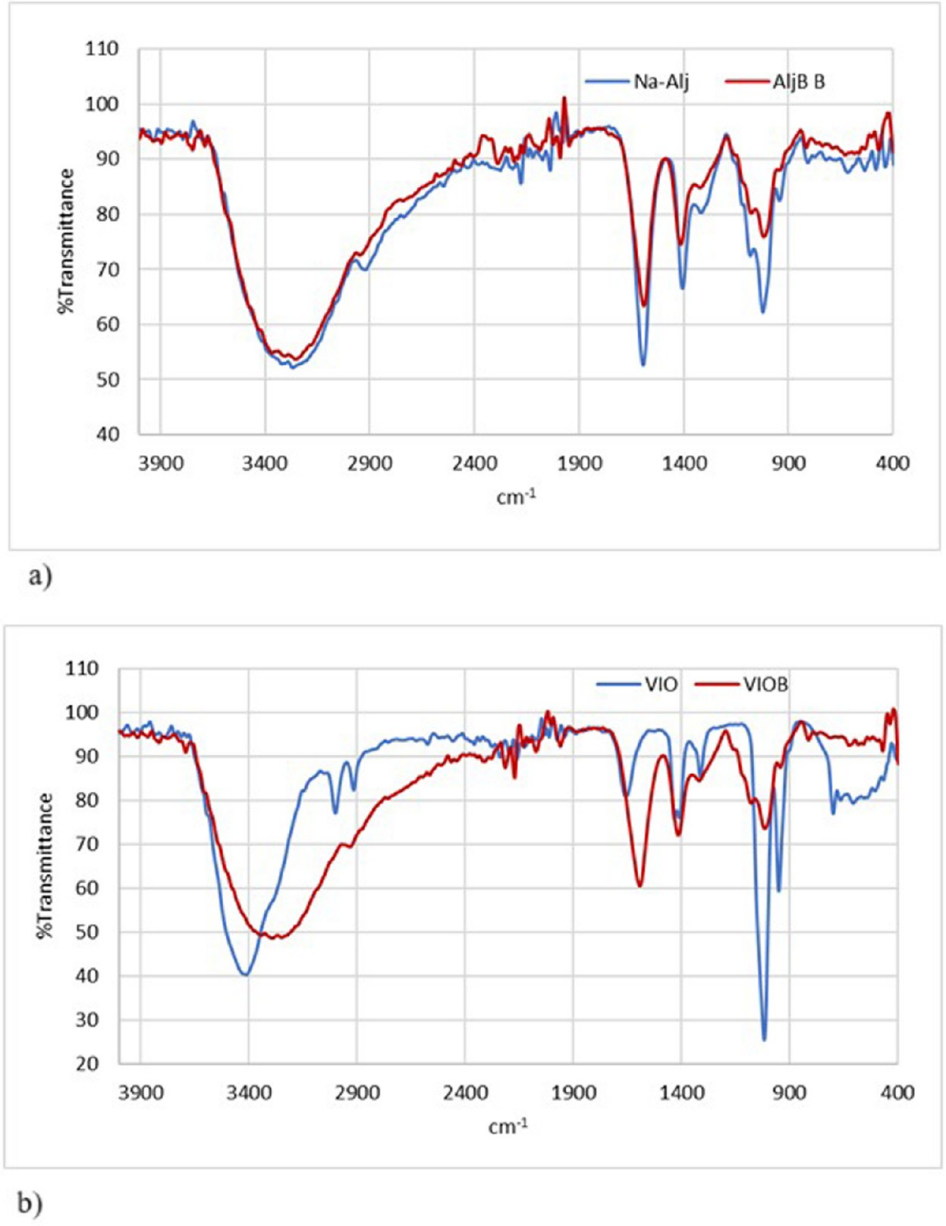
FTIR spectra of alginate and alginate beads (a), and violacein and violacein/alginate beads (b).

### Swelling Ratio and In Vitro Release Analysis of Violacein-Alginate Beads

The swelling properties of beads were examined by measuring water uptake over time. Alginate and violacein-alginate beads were placed in acidic (pH 1.2) and basic (pH 7.4) buffer solutions, and their swelling behavior was plotted as a function of time.

The swelling percentages at different pH levels are shown in Figure 5 **a-b**. The ionic nature of alginate facilitates pH-dependent degradation.^38^ Violacein-alginate beads exhibited higher swelling at pH 7.4 than at pH 1.2, consistent with previous studies.^39,40^ At pH 1.2, alginate beads reached an equilibrium swelling degree of 110%, while violacein-alginate beads exhibited 106% swelling. At pH 7.4, both types of beads reached 180% swelling within 2 h, demonstrating no dissolution over the observed period.

**Figure 5:**
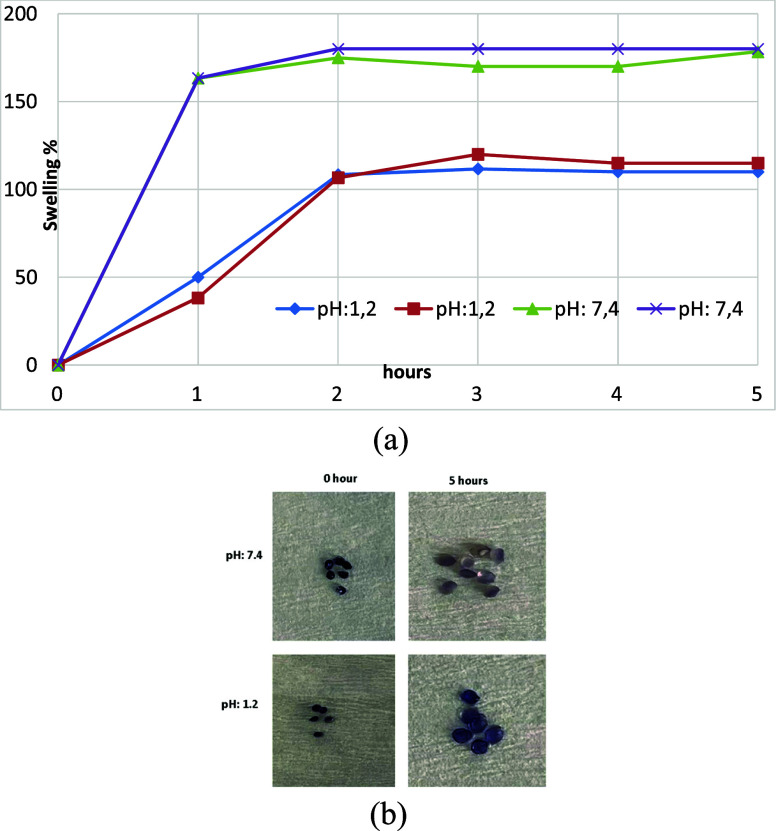
(a) Optical microscopy images of violacein-alginate beads before and after swelling and (b) swelling kinetics of dry violacein-alginate beads in simulated gastric fluid (SGF, pH 1.2) and simulated intestinal fluid (SIF, pH 7.4).

The drug release profile of violacein-alginate beads was evaluated in simulated digestive conditions (pH 1.2 and pH 7.4) over an 8 h period. Samples (1 mL) were taken hourly, and violacein concentrations were measured spectrophotometrically. As shown in Figure 6, over 50% of violacein was released at pH 7.4 within 1 h, reaching nearly 100% after 2 h. At pH 1.2, only ∼ 55% of violacein was released within the first 2 h. By the end of the experiment (8 h), cumulative release reached ∼ 75% and ∼ 100% under simulated gastric and intestinal conditions, respectively.

**Figure 6:**
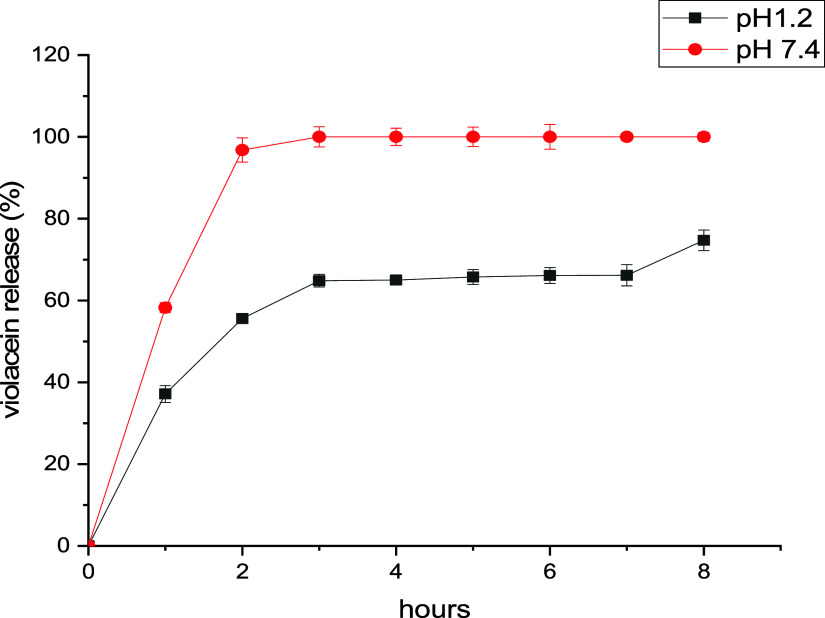
In vitro release profile of violacein from violacein-alginate beads in simulated gastrointestinal fluids (pH 1.2–7.4). Error bars represent standard deviation (*n* = 3).

### Antibacterial and Antibiofilm Properties of Violacein-Alginate Beads

The antibacterial properties of sodium alginate and violacein-alginate beads (5–10 mg) against *S. aureus* were evaluated using the well diffusion method. No inhibition zones were observed (Figure 7a). Cell count analysis (20–1 mg/mL) also indicated no reduction in the viability of *S. aureus.*

**Figure 7:**
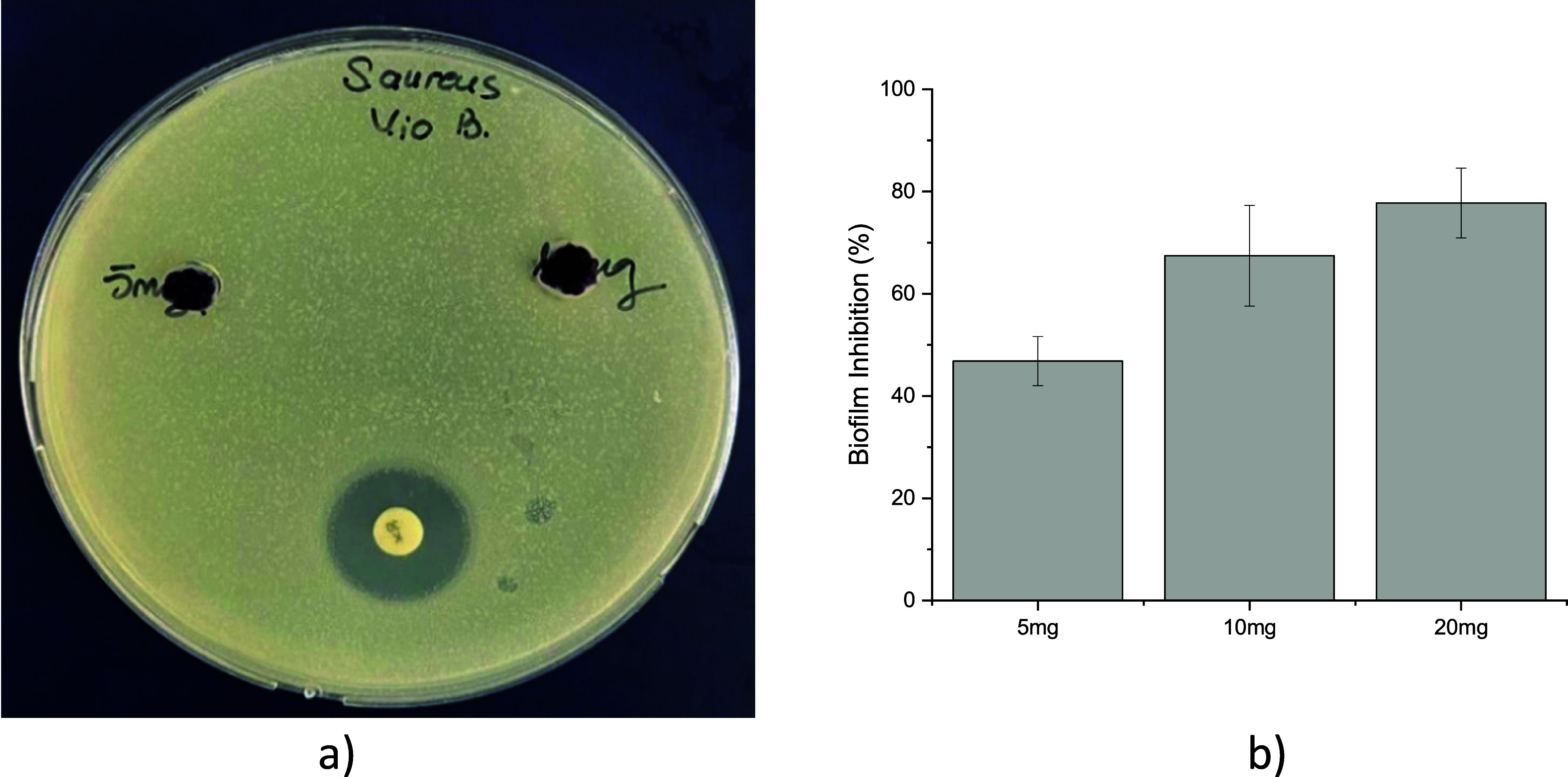
(a) Agar well diffusion assay of violacein-alginate beads (10 mg and 20 mg) against *Staphylococcus aureus*. K: Kanamycin positive control (30 μg/mL). (b) Quantitative analysis of *S. aureus* biofilm inhibition by violacein-alginate beads (5, 10, and 20 mg/mL) using the crystal violet assay. Inhibition rates were 46.8 ± 4.8, 67.4 ± 9.9, and 77.4 ± 6.8% for 5, 10, and 20 mg/mL, respectively. Data represent mean ± SD from three independent experiments.

Bacterial biofilms exhibit pathogenic properties, are commonly associated with chronic and hospital-acquired infections, and their formation significantly increases bacterial resistance, posing challenges in the elimination of microbial infections.^41,42^ Conventional antibiotics often show limited effectiveness in penetrating biofilms or eliminating dormant persister cells, which contributes to the persistence and recurrence of infections.^43^ The development of systemic or topical antibiofilm agents could significantly improve the effectiveness of traditional antimicrobial therapies.^44,45^ In this study, violacein-alginate beads (5, 10, and 20 mg/mL) exhibited notable antibiofilm activity, achieving inhibition rates of 46.8 ± 4.8%, 67.4 ± 9.9%, and 77.4 ± 6.8%, respectively (Figure 7b), without compromising the viability of *Staphylococcus aureus*. These results indicate that violacein-alginate beads effectively inhibit biofilm formation in a dose-dependent manner.

### Molecular Docking

Molecular docking is a computational approach employed to predict the binding interactions between a receptor (e.g., protein or nucleic acid) and a ligand (small molecule).^46^ This method calculates a binding score, representing the estimated change in free energy (ΔG) upon ligand–receptor complex formation. The scoring function incorporates critical factors such as hydrogen bonding, steric constraints, and metal ion coordination. A more negative score correlates with higher binding affinity.^47^

Computational tools are indispensable for elucidating biofilm formation mechanisms and identifying potential antibiofilm compounds from large-scale databases. Such agents can selectively target biofilm-associated proteins and modulate their activity, as validated by *in vitro* studies.^48–52^

*S. aureus*, a prevalent opportunistic pathogen, is a major causative agent of skin, soft tissue, and implant-associated infections. Its virulence is exacerbated by biofilm formation, which initiates with bacterial adhesion to host tissues or abiotic surfaces, followed by cell aggregation and proliferation within a protective extracellular polymeric matrix (EPS). Biofilms confer enhanced resistance to antibiotics and environmental stressors, making their disruption a promising strategy to combat antimicrobial resistance.^49–52^ The *icaADBC* operon orchestrates biofilm development by regulating polysaccharide intercellular adhesin (PIA) production. It encodes three membrane-integrated proteins (IcaA, IcaD, IcaC) and one extracellular protein (IcaB), all critical for EPS assembly. Additionally, surface-associated proteins contribute to biofilm maturation.^49,50^

In this study, we performed molecular docking and dynamics simulations to evaluate violacein’s binding interactions with IcaA, IcaB, IcaC, and IcaD. The calculated binding free energies were – 10.6 kcal/mol (IcaA), – 8.0 kcal/mol (IcaB), – 11.2 kcal/mol (IcaC), and – 9.0 kcal/mol (IcaD), demonstrating strong affinity between violacein and the *icaADBC*-encoded proteins. Notably, violacein exhibited the highest stability with IcaC (−11.2 kcal/mol).

Violacein’s high binding affinity toward biofilm-related proteins suggests its potential as an antibiofilm agent. Interaction analyses revealed that van der Waals forces dominated stabilization across all complexes, supplemented by conventional hydrogen bonds, π-π stacking, and π-alkyl interactions. Detailed binding modes are illustrated in Figures 8–9

**Figure 8:**
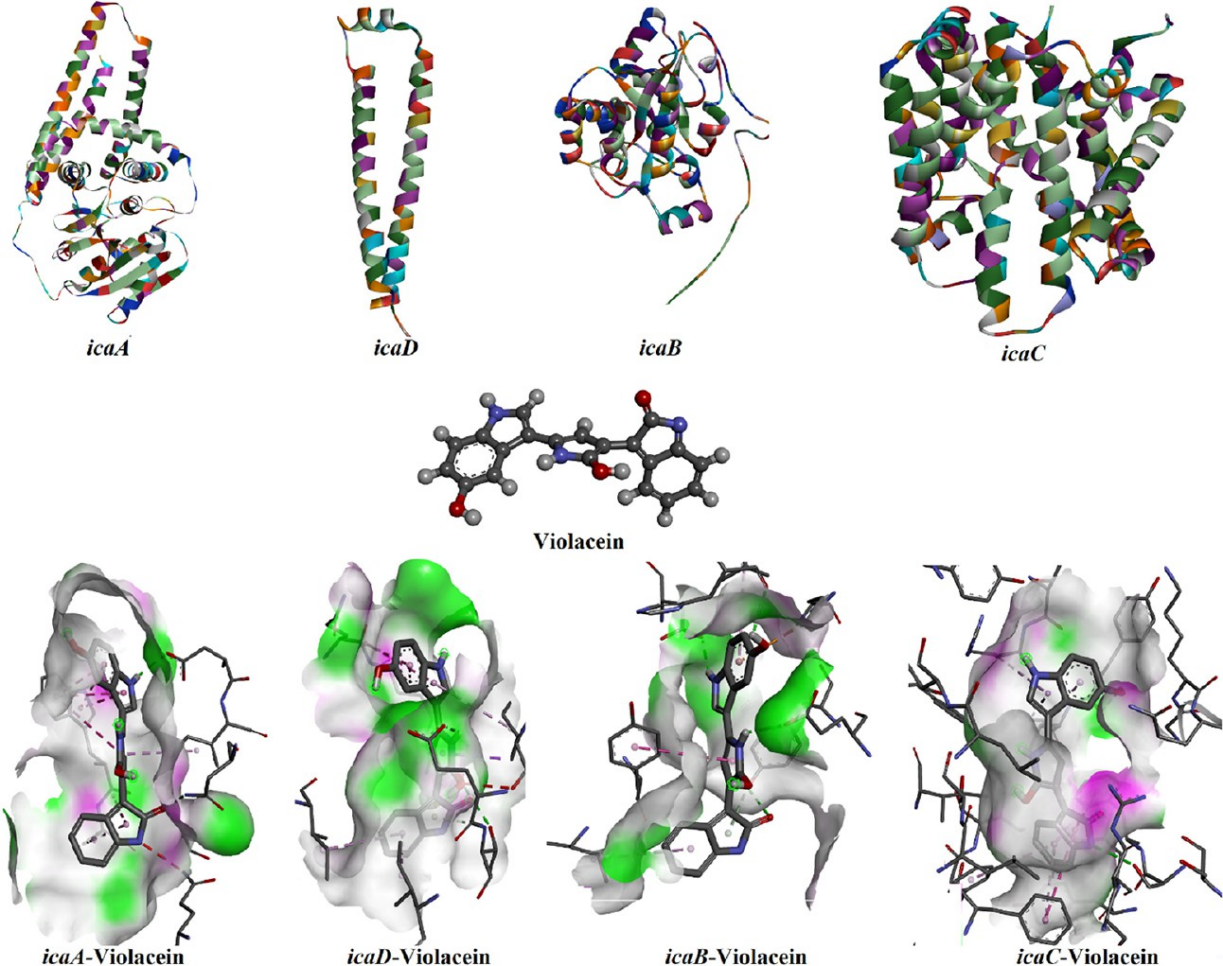
Molecular docking analysis: Three-dimensional structural representations of *S. aureus* biofilm-related proteins (icaA, icaB, icaC, icaD; UniProt IDs: Q9RQP9, Q9RQP8, Q9RQP7, and Q9RQP6), violacein, and protein–ligand binding conformations with interaction details.

**Figure 9:**
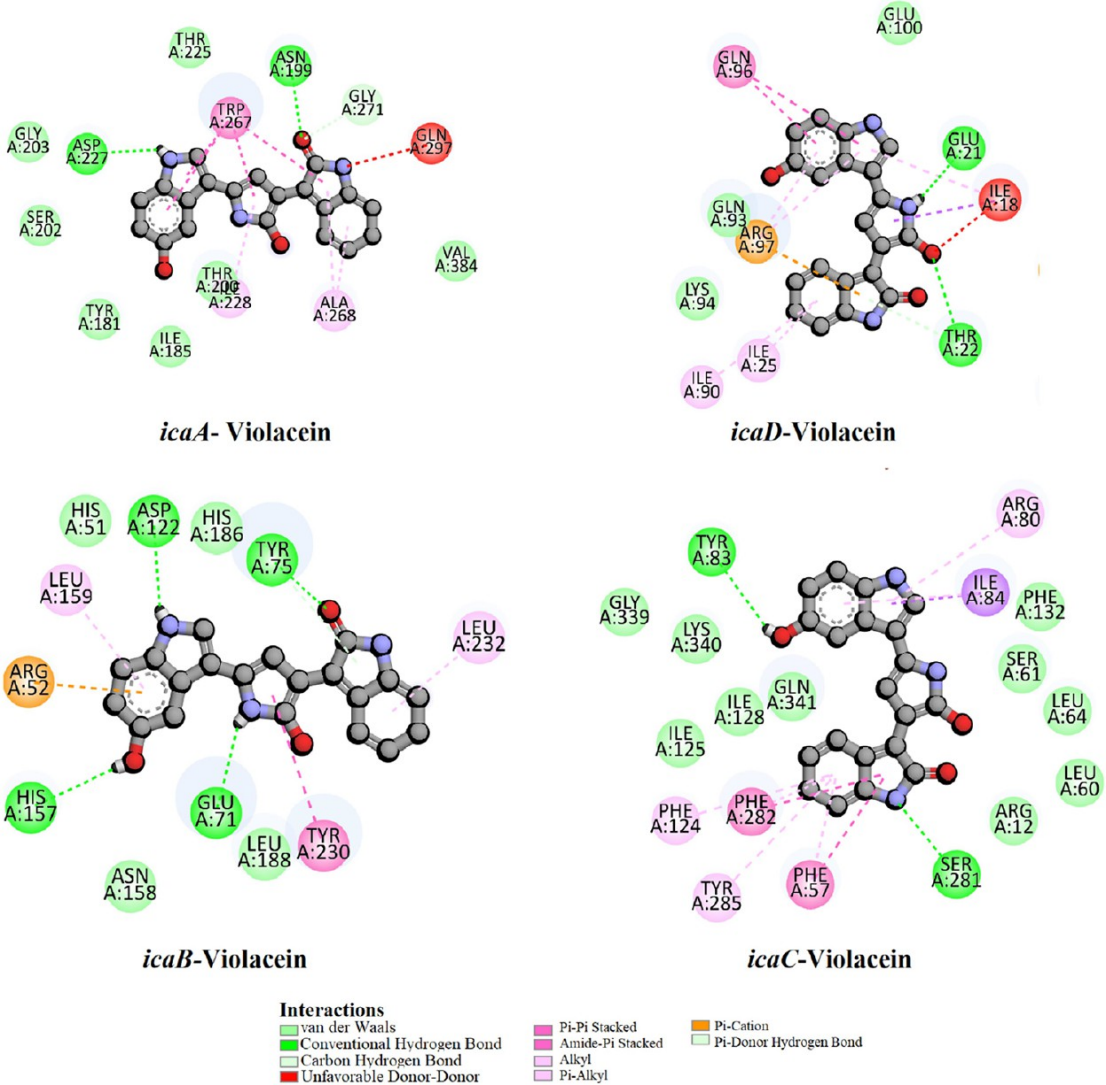
Two-dimensional interaction diagrams of violacein with *S. aureus* biofilm-related proteins (UniProt: icaA/Q9RQP9, icaB/Q9RQP8, icaC/Q9RQP7, and icaD/Q9RQP6).

IcaC exhibited the strongest binding affinity with a docking score of – 11.2 kcal/mol, suggesting a highly stable ligand–receptor interaction. The ligand formed extensive interactions with hydrophobic residues such as PHE124, PHE132, PHE280–PHE283, TYR285, and aromatic/hydrophilic residues like LYS2, ARG5, and GLN275, indicating a combination of π–π stacking, hydrogen bonding, and van der Waals forces in the binding pocket. IcaA also showed notably strong binding (−10.6 kcal/mol). The interaction profile included multiple hydrophobic and polar residues such as TRP18, TYR181, GLY203, ARG264, TRP303, TYR310, and TRP377, implying that the ligand fits well into a structurally complex and residue-rich binding cavity. IcaD demonstrated a moderate binding affinity of – 9.0 kcal/mol, with interactions mainly involving polar residues (e.g., GLN93, ARG97) and hydrophobic residues (ILE18, LEU24, PHE85–PHE86), supporting the ligand’s moderate binding stability. IcaB displayed the lowest binding affinity among the four, with a docking score of – 8.0 kcal/mol. Nevertheless, it still established relevant interactions, notably with HIS51, ARG54, GLU125, and TYR230, indicating a potential, albeit less stable, binding mode compared to the others. These results suggest that the ligand has the highest binding potential against IcaC and IcaA, highlighting these proteins as the most promising targets for interfering with the *ica*-mediated biofilm pathway. The extensive interaction with aromatic and polar residues in these proteins supports a favorable orientation and strong molecular recognition within the active site, which could translate into significant inhibitory activity. Details of molecular docking interactions between protein-violacein complexes are provided in the supplementary file (Table S1).

Given biofilms’ established role in treatment-resistant infections, these *in silico* results indicate violacein’s capacity to interfere with critical biofilm developmental pathways through targeted protein interactions. The robust docking scores provide compelling computational evidence for violacein’s antibiofilm potential, meriting comprehensive *in vitro* and *in vivo* validation studies.

### Drug Likeness Properties

The harmony between a drug’s physicochemical characteristics and its basic biopharmaceutical features within the human body is generally referred to as “drug-likeness”.^53^ A pivotal framework for assessing the potential of an active substance as a drug candidate is provided by Lipinski’s Rule of Five. This rule enumerates several essential requirements: The requirements listed below must be fulfilled: (i) less than 500 Da of molecular weight; (ii) significant lipophilicity; (iii) no more than five hydrogen bond donors or acceptors; and (v) a molar refractivity between 40 and 130.^54^ A compound is generally considered drug-like if it meets at least two of these criteria, which serves as a useful filter in the screening of ligands for drug-like properties. As demonstrated in Table 2, the violacein molecule fulfills Lipinski’s Rule of Five., confirming its potential drug-likeness.

**Table 2:** Predicted Physicochemical Properties, Lipophilicity, and Drug Likeness of Violacein

parameters	violacein
Properties of Physicochemistry	
formula	C_20_H_13_N_3_O_3_
molecular weight g/mol	343.34 g/mol
number of heavy atoms	26
number of aromatic heavy atoms	5
number of rotatable bonds	2
hydrogen bond acceptor (HBA)	3
hydrogen bond donors (HBD)	4
molar refractivity	100.20
topological polar surface area (TPSA)(Å^2^)	101.47
Lipophilicity	
log*P*_o/w_(iLOGP)	1.65
log*P*_o/w_(XLOGP3)	2.74
log*P*_o/w_(WLOGP)	1.56
log*P*_o/w_(MLOGP)	1.56
log*P*_o/w_(SILICOS-IT)	4.65
consensus log*P*_o/w_	2.43
Drug Likeness	
violation of Lipinski’s rule of five	suitable
bioavailability score	0.56

There are six hydrogen bond donors and four hydrogen bond acceptors in the violacein molecule. At 343.34 g/mol, its molecular weight fulfills Lipinski’s Rule of Five. Additionally, it has been found that violacein has a Log P value of 2.43, which indicates the compound’s lipophilicity and capacity to traverse biological membranes. The molar refractivity of violacein was determined to be 100.2, well within the acceptable range of 40 to 130. The bioavailability score for violacein was 0.56. The presence of two rotatable bonds in the molecule suggests some structural flexibility. Furthermore, a drug candidate’s optimal topological polar surface area (TPSA) is normally less than 120 Å^2^. And violacein’s TPSA was calculated to be 101.47 Å^2^, suggesting favorable intestinal absorption. A comprehensive analysis of the parameters in Table 2 confirms that the active violacein molecule falls within the acceptable ranges for drug-likeness.

#### ADMET Predictions

The process of finding and developing new drugs heavily relies on the prediction of ADMET (absorption, distribution, metabolism, excretion, and toxicity) features. To help researchers design and optimize pharmaceutical molecules, in silico ADMET evaluation models have been created.^55^ W. Bhat et al. included some basic ADMET parameters in their research on the effects of violacein on epidermal growth factor.^56^ In their study, Verma and Pandey explored the pharmacological properties of violacein in the context of its effects against ischemic stroke.^57^ However, no comprehensive study has been identified that covers all physicochemical properties, absorption, distribution, metabolism, excretion, and toxicity parameters of violacein. In this study, the ADMET analysis serves as a complementary contribution to the literature, providing researchers with an integrated source encompassing all ADMET parameters.

Table 3 outlines the ADMET characteristics of violacein molecules. Human intestinal absorption (HIA) levels and Caco-2 permeability are important markers of a drug’s capacity for intestinal absorption. For these metrics, the suggested threshold values are 30% for HIA and 0.90 for Caco-2 permeability, respectively.^53^ For violacein, these values were found to be 20.56 and 86.35%, respectively. The aqueous solubility of a drug, which is influenced by the hydrogen bonding capacity of its functional groups with water, plays a significant role in the drug’s absorption and its ability to reach the target site. Violacein molecules exhibited high aqueous solubility. A drug’s capacity to permeate organs and tissues is indicated by its steady-state volume of distribution (VDss); Widespread distribution is indicated by VDss values greater than 0.112 in the literature.^58^ The VDss value of 0.49 L/kg indicates that violacein exhibits a moderate distribution, with a tendency to remain primarily within the systemic circulation rather than extensively penetrating peripheral tissues. This pharmacokinetic characteristic may influence its tissue-specific efficacy and bioavailability, particularly in compartments requiring higher distribution volumes. The Blood–Brain Barrier (BBB+) value represents the compound’s potential to penetrate the blood–brain barrier and is expressed as a normalized probability score ranging from 0 to 1. A value between 0.00–0.30 indicates poor or negligible BBB permeability, 0.30–0.70 reflects moderate permeability potential, and values above 0.70 suggest high permeability.^59^ The observed BBB+ value of 0.54 implies that the compound may possess a moderate ability to cross the blood–brain barrier.

**Table 3:** ADMET Properties Predicted for the Violacein Molecule

Parameters	Violacein
**Absorption**	
Caco-2 cell permeability (nm/s)	20.558
MDCK cell permeability (nm/s)	7.33
*P*-glycoprotein substrate	0.46
human intestinal absorption (HIA + , %)	86.35
pure water solubility (mg/L)	5.04
plasma protein binding (PPB, %)	98.25
blood–brain barrier (BBB + )	0.54
volume distribution (VDss)	0.495
**Metabolism**	
CYP1A2	yes
CYP2C9 inhibition	no
CYP2C19 inhibition	no
CYP2D6 inhibition	no
CYP2D6 substrate	no
CYP3A4 inhibition	no
log*K*_p_ (skin permeation cm/s)	–6.45
**Excretion**	
plasma clearance (CLp)	0.72
renal clearance (CLr)	0.669
half-life	1.28
mean retention time (MRT)	1.35
**Toxicity**	
human ether-a-go-go-related gene (HERG) inhibition (%)	41.94
maximum recommended daily dose (FDAMDD) (%)	0.91
drug-induced liver injury (DILI)	49.83
Ames toxicity	44.05
rat oral acute toxicity	0.75
skin reaction	0.60
eye corrosion	0.001
minnow toxicity	0.004
carcinogens	0.604

hERG channels are potassium channels that regulate cardiac rhythm, and their inhibition is associated with the risk of cardiotoxicity.^60^ An inhibition rate of 41.94% indicates a moderate level of hERG blockade, suggesting that the molecule may pose a potential arrhythmogenic risk. This finding should be carefully considered in the evaluation of the compound as a drug candidate. The plasma protein binding (PPB) value of 98.25% indicates that the compound binds extensively to serum proteins, primarily albumin. As a result, only a small fraction remains pharmacologically active in the free form. This high PPB may influence drug distribution, prolong half-life, and increase the risk of drug–drug interactions, necessitating careful consideration in multidrug regimens or in patients with altered protein levels. The DILI (Drug-Induced Liver Injury) score represents the likelihood that a compound may cause liver injury as a result of drug exposure.^61^ A DILI probability of 49.83% suggests a moderate risk of hepatotoxicity. While values exceeding 50% typically raise significant safety concerns, violacein’s score, being near this threshold, implies a potential for the formation of reactive metabolites or direct hepatocellular toxicity during hepatic metabolism. This finding underscores the necessity for close monitoring of liver function parameters throughout the clinical development process.

## Conclusions

In this study, violacein was successfully encapsulated within biodegradable, nontoxic, biocompatible, and pH-responsive alginate beads. The violacein-alginate beads (VIABs) demonstrated significant antibiofilm activity, effectively inhibiting *Staphylococcus aureus* biofilm formation. Molecular docking analysis demonstrated violacein’s strong binding affinity with key *S. aureus* target proteins, providing mechanistic insight into its antibiofilm effects. Additionally, the formulation facilitated controlled violacein release under simulated gastrointestinal conditions, mitigating abrupt alkaline degradation and improving stability.

These findings underscore the potential of VIABs as a promising therapeutic agent for medical applications, such as antimicrobial treatments, and industrial uses, including food preservation. By combining violacein’s potent bioactivity with the alginate delivery system, this study presents a viable strategy to enhance violacein’s solubility and bioavailability, addressing a critical limitation in its practical application.

## Methods

### Bacterial Strains

*S. aureus* ATCC 25923 and *C. violaceum* ATCC 12472 strains were obtained from the Department of Biology, Süleyman Demirel University (Isparta, Turkey). All bacterial strains were cultured in Luria–Bertani (LB) broth or on LB agar plates at 37 °C for 24 h and stored at 4 °C for further use.

Ethanol (Merck), DMSO (Merck), Luria–Bertani Broth (Miller), and Luria–Bertani Agar (Miller) were used in this study.

### Extraction of violacein pigment from *Chromobacterium violaceum* ATCC 12472

*C. violaceum* ATCC 12472 was incubated in LB liquid medium at 120 rpm and 36 °C for 24 h. After incubation, the culture was centrifuged at 15,000 rpm, and the supernatant was discarded. To the pellet at the bottom of the tube, 1 mL of ethanol was added and left for 3 min in an ultrasonic bath (Bandelin, Sonorex, RK-100), followed by centrifugation (15000 rpm), and the supernatant was transferred to new tubes. The extraction process was repeated five times, with a total ultrasonic bath time of 15 min and 5 mL ethanol addition each time. The solvent in the resulting mixture was evaporated in a rotary evaporator (at 50 °C). The obtained pigment was then filtered through a 0.45 μm PVDF syringe filter into dimethyl sulfoxide (DMSO) and stored at +4 °C for analysis. The absorbance of the resulting DMSO-VIO mixture was measured at OD585 nm using a UV spectrophotometer.^39^

### Determination of Antibacterial Activity and Minimum Inhibitory Concentration (MIC) Values of Violacein Extract

The antibacterial effect of VIO (25–200 μg/mL) against *S. aureus* was tested using the well diffusion method. After incubation, the diameters of the inhibition zones around the wells were measured to evaluate their antibacterial effects. Each sample was tested at least three times, and mean and standard deviation values were calculated.

The MIC of the VIO pigment against *S. aureus* was determined using the tube dilution method.^62^ VIO samples within the range of 25–200 μg/mL were added to 1 mL LB broth. Subsequently, 20 μL of bacterial cultures (0.5 McFarland) was added to the mixture, and it was incubated at 36 °C for 24 h. The MIC was defined as the lowest concentration that completely inhibited bacterial growth, with the presence of only a few colonies.

### Evaluation of the Effect of Violacein Extract On Cell Numbers

To determine the reduction in cell numbers caused by VIO in *S. aureus* the drop method was used.^63^

For this purpose, VIO at concentrations determined as MIC and MIC/2 for *S. aureus* was added to 1 mL of 0.85% NaCl solution. Then, 20 μL of bacterial cultures (0.5 McFarland) was added, and the mixture was incubated at 36 °C for 24 h. At the end of the incubation period, serial dilutions were prepared from samples containing and not containing VIO, and 5 μL from each dilution was dropped onto LB agar plates and incubated at 36 °C for 24 h. After incubation, the logarithmic reduction in cell numbers of samples containing and not containing VIO was evaluated.

### Investigation of the Biofilm Inhibition Properties of Violacein Extract

A static biofilm formation assay was performed to examine violacein’s effect on *S. aureus* using the crystal violet staining method.^64^

The experiment was performed in triplicate, and the mean ± standard deviation (SD) was calculated. The percentage of biofilm inhibition was calculated according to

Inhibition%=100−((Abs570nmSample/Abs570nmcontrol)×100)

### Preparation and Physicochemical Characterization of Sodium Alginate and Violacein Loaded Sodium Alginate Beads

Alginate and VIO-loaded alginate beads were synthesized via the ionic gelation technique.^34^ Initially, 20 mL of 3% (w/v) alginate aqueous solution was stirred in a magnetic stirrer at 40 °C and 400 rpm for 30 min to form a suspension. The room temperature was maintained at approximately 25 °C. The prepared solution was then dropped into a 2.5% (w/v) CaCl_2_ aqueous solution using an insulin syringe (0.30 × 8 mm) to induce bead formation. The syringe height was fixed at 20 cm. These beads were further stirred in the CaCl_2_ solution for approximately 15 min using a magnetic stirrer. Subsequently, the beads were washed with distilled water, dried at 30 °C in an oven for 24 h, and stored accordingly.

For the preparation of violacein-alginate beads, the same procedure was followed with the addition of 400 μL (10 mg/mL) of violacein extract after the suspension formation, followed by further stirring for 60 min. The subsequent steps were identical to the preparation of alginate beads.

The particle size of the synthesized alginate beads was analyzed using the ImageJ software, and the results were expressed as the average diameter (μm).^39^ Measurements were repeated at least three times for 100 beads each time, and the mean and standard deviation values were calculated using ImageJ.

### SEM Analysis of Alginate and Violacein-Alginate Beads

SEM analyses of violacein-alginate beads were conducted at the Innovative Technologies Application and Research Center (YETEM) of Süleyman Demirel University using a FEI Quanta FEG 250/EDAX-EDS scanning electron microscope. The samples were mounted with double-sided carbon tape for examination, and no coating was applied before imaging.

### FTIR Analysis of Alginate and Violacein-Alginate Beads

The structural analysis of violacein-alginate beads was performed at the Innovative Technologies Application and Research Center (YETEM) of Süleyman Demirel University by obtaining FTIR spectra. Sample analyses were conducted using a PerkinElmer spectrum bx spectrophotometer with ATR technique at a resolution of 4 cm-1 and scanning from 4000 to 400 cm-1 in the mid-infrared region with 16 scans.

### Swelling Ratio and In Vitro Release Analysis of Alginate and Violacein-Alginate Beads

The swelling analysis of beads was performed using the gravimetric method by adding 20 mg of beads to 40 mL of pH 1.2 and pH 7.4 PBS at 36 °C.^65^ The weights of beads were measured hourly until they reached maximum swelling. The percentage swelling capacity was calculated using Equation $S\left(\%\right)=\left(\frac{m1-m2}{m2}\right)\times 100$ , where m1 represents the weight of dry beads and m2 represents the weight of swollen beads.

The violacein release from violacein-alginate beads was determined by measuring absorbance using a UV-1601 UV Visible Spectrophotometer (Shimadzu) at λmax 585 nm.^66^ In vitro release studies were conducted in PBS buffers with pH 7.4 and 1.2 at 36 °C. Beads were suspended in 5 mL of PBS buffer. The absorbance of solutions was measured at specified intervals (1, 2, 4, 6, 8, and up to 24 h) at 585 nm. The study was repeated at least three times, and mean and standard deviation values were calculated.

### Antibacterial and Antibiofilm Properties of Violacein-Alginate Beads

The antibacterial effects of all prepared beads on *S. aureus* was determined using the well diffusion test method.^67^ All measurements were repeated at least three times, and mean and standard deviation values were calculated.

The antibiofilm effects of violacein-alginate beads on *S. aureus* was determined using the crystal violet assay.^68^ The study was repeated at least three times, and mean and standard deviation values were calculated.

### Computational Details and Molecular Docking Analysis

High-resolution crystal structures of the target proteins (UniProt accession numbers: Q9RQP9, Q9RQP8, Q9RQP7, and Q9RQP6) were retrieved from the UniProt Data Bank. Molecular docking simulations were conducted using AutoDock Vina.^69^ Discovery Studio Visualizer application was used to visualize receptor–ligand interactions (2016). ADMET properties were predicted using the preADMET and SwissADME web servers (https://preadmet.qsarhub.com/ and http://www.swissadme.ch/).

### Statistical Analyses

All data were analyzed using Origin 2019 software. Data are presented as mean ± standard deviation (SD) from triplicate experiments. Error bars define the SD of triplicate values.

## Supplementary Material



## Notes

High-performance liquid chromatography (HPLC) analysis of violacein extract and molecular docking interactions between different protein-violacein complexes (PDF)
