# Supplementary material for: Molecular Docking and In Vitro Evaluation of Violacein-Alginate Beads for Targeted Inhibition of Staphylococcus aureus Biofilm Formation
Source: ACS Omega. 2025 Jul 16;10(29):31827–39. doi: 10.1021/acsomega.5c03106 (PMC12311687; doi:10.1021/acsomega.5c03106)
Supplement: Supplementary file 1 [file ao5c03106_si_001.pdf]

Molecular Docking and In Vitro Evaluation of Violacein-Alginate Beads for Targeted Inhibition of *Staphylococcus aureus* Biofilm Formation

Çağdaş Deniz PERİZ[1], Seyhan ULUSOY[1], Neslihan KAYA KINAYTÜRK[2]*

Çağdaş Deniz PERİZ[1], denizperiz@gmail.com

Seyhan ULUSOY [1*], (seyhanulusoy@sdu.edu.tr)

Neslihan KAYA KINAYTÜRK[2], (nkinayturk@mehmetakif.edu.tr)

[1]Süleyman Demirel University, Faculty of Engineering and Natural Sciences, Biology Department, 32260 Isparta, Türkiye.

[2]Mehmet Akif Ersoy University, Faculty of Arts and Science, Nanoscience and Nanotechnology Department, 15100 Burdur, Türkiye.

* Corresponding author: seyhanulusoy@sdu.edu.tr

High-performance liquid chromatography (HPLC) analysis of violacein extract

The crude violacein extract was analyzed using a Shimadzu HPLC system. Chromatographic separation was performed on an Agilent Zorbax XDB-C18 reversed-phase column (5 µm particle size, 4.6 mm inner diameter × 250 mm length) maintained at 30°C. The isocratic mobile phase consisted of methanol-water (70:30, v/v), at a flow rate of 1 mL/min. Violacein was monitored at 585 nm[1].

The HPLC chromatogram demonstrating the separation profile of the violacein extract is provided in Figure **S1**.

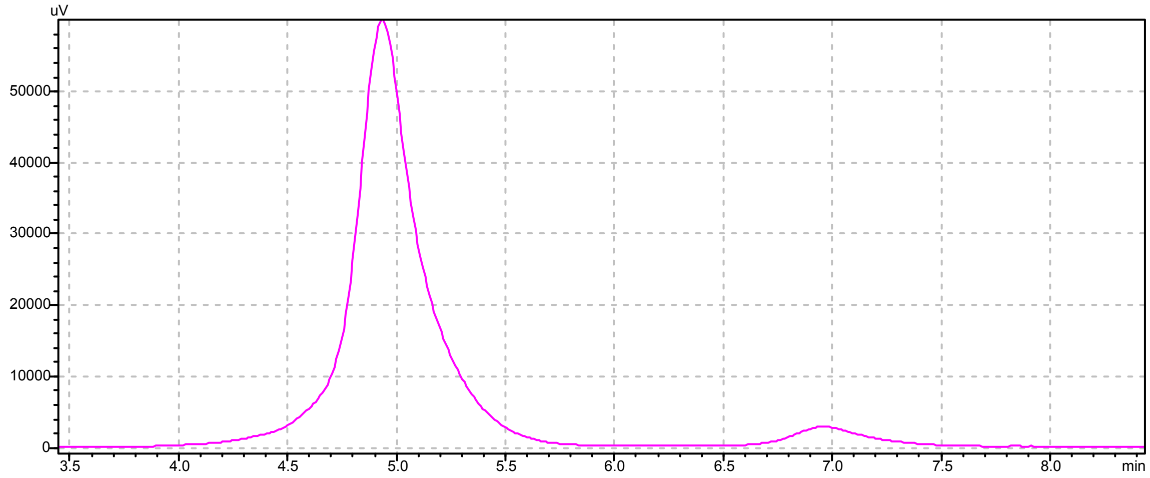

Figure S1. HPLC chromatogram of the violacein extracted from *Chromobacterium violaceum.*

Table S1. Molecular docking interactions between different protein-violacein complexes.

PDB ID	Binding affinity (Kcal/mol)	Interacted Residues
**Ica A**	-10.6	LEU7 LEU8 TYR10 TRP18 TYR181 ILE185 ILE188 LYS189 GLN192 ASN199 THR200 ILE201 SER202 GLY203 VAL204 THR225 ASP227 ILE228 ARG264 VAL265 TRP267 ALA268 GLN269 GLY270 GLY271 HIS272 LEU293 GLU296 GLN297 SER300 ILE301 TRP303 VAL304 VAL307 TYR310 LEU311 MET340 THR341 TRP371 THR374 TYR376 TRP377 ILE379 ASN380 ALA381 VAL383 VAL384
**Ica B**	-8.0	Chain A: HIS51 ARG52 ARG54 LYS55 ASN57 ASN60 ILE63 GLU71 ILE72 TYR75 ASP122 ASP124 GLU125 THR126 GLU129 ASN130 ILE147 ASN155 HIS157 ASN158 LEU159 ASP160 MET161 HIS186 ASN187 LEU188 ASN192 TYR230 LEU232
**Ica C**	-11.2	LYS2 LYS3 ILE4 ARG5 LEU8 VAL9 ARG12 PHE57 ILE58 LEU60 SER61 LEU63 LEU64 THR65 LEU67 ASN68 TYR69 TYR76 THR79 ARG80 TYR83 ILE84 PHE124 ILE125 ILE128 PHE132 GLN275 SER278 ALA279 PHE280 SER281 PHE282 PHE283 TYR285 GLY339 LYS340 GLN341 LYS344 LEU345
**Ica D**	-9.0	SER14 ASN17 ILE18 VAL19 ARG20 GLU21 THR22 LEU24 ILE25 CYS29 PHE83 PHE85 PHE86 THR87 SER89 ILE90 LEU91 ILE92 GLN93 LYS94 GLN96 ARG97 GLU100

Reference

(1) Gıda, A.; Deniz Periz, Ç.; Ulusoy, S.; Tınaz, G.; Şekerler, T. Antibacterial and Anticancer Activities of Violacein Extracted Through Ultrasound-Assisted Extraction Method. *Akademik Gıda* 2020, *18* (3).
